# Radiation exposure during retrograde intrarenal surgery (RIRS): a prospective multicenter evaluation

**DOI:** 10.1007/s00345-020-03160-9

**Published:** 2020-03-21

**Authors:** Simon Hein, Konrad Wilhelm, Arkadiusz Miernik, Martin Schoenthaler, Rodrigo Suarez-Ibarrola, Christian Gratzke, Johannes Salem, Leonidas Karapanos, Christopher Netsch, Benedikt Becker, Armin Secker, Julian Veser, Andreas Neisius, Hans-Martin Fritsche, Marco Julius Schnabel

**Affiliations:** 1 Department of Urology, Medical Center-University of Freiburg (institution to which this work is attributed), Faculty of Medicine, University of Freiburg, Hugstetter Str. 55, 79106 Freiburg, Germany; 2 Department of Urology and Robot-Assisted and Reconstructive Surgical Urology, University of Cologne, Cologne, Germany; 3 Department of Urology, Asklepios Hospital Barmbek, Rübenkamp 220, 22291 Hamburg, Germany; 4 Department of Urology, Medical Center, University of Muenster, Albert-Schweitzer-Campus 1, A1, 48149 Muenster, Germany; 5 Department of Urology, General Hospital Vienna, Medical University Vienna, Waehringer Guertel 18-20, 1090 Vienna, Austria; 6 Department of Urology, Krankenhaus der Barmherzigen Brüder Trier, Johannes Gutenberg University Mainz, Trier, Germany; 7 Department of Urology, Chirurgische Klinik München-Bogenhausen, Munich, Germany; 8 Department of Urology, Caritas St. Josef Medical Centre, University of Regensburg, Landshuter Str. 65, 93053 Regensburg, Germany

**Keywords:** Flexible ureteroscopy, Fluoroscopy, Ionizing radiation exposure, X-ray exposure, Nephrolithiasis, Urolithiasis

## Abstract

**Purpose:**

Retrograde intrarenal surgery (RIRS) may require extensive X-ray usage. We evaluated the impact of preoperative surgeon briefing regarding the inclusion and evaluation of fluoroscopy time (FT) and dose area product (DAP) in a multicenter study on the applied X-ray usage.

**Methods:**

A prospective multicenter study of 6 tertiary centers was performed. Each center recruited up to 25 prospective patients with renal stones of any size for RIRS. Prior to study´s onset, all surgeons were briefed about hazards of radiation and on strategies to avoid high doses in RIRS. Prospective procedures were compared to past procedures, as baseline data. FT was defined as the primary outcome. Secondary parameters were stone-free rate (SFR), complications according to the Clavien, SATAVA and postureteroscopic lesion scale. Results were analyzed using *T* test, chi-squared test, univariate analysis and confirmed in a multivariate regression model.

**Results:**

303 patients were included (145 retro- and 158 prospective). Mean FT and DAP were reduced from 130.8 s/565.8 to 77.4 s/357.8 (*p* < 0.05). SFR was improved from 85.5% to 93% (*p* < 0.05). Complications did not vary significantly. Neither stone position (*p* = 0.569), prestenting (*p* = 0.419), nor surgeons’ experience (> 100 RIRS) had a significant impact on FT. Significant univariate parameters were confirmed in a multivariate model, revealing X-ray training to be radiation protective (OR − 44, *p* = 0.001).

**Conclusions:**

Increased surgeon awareness of X-ray exposure risks has a significant impact on FT and DAP. This “awareness effect” is a simple method to reduce radiation exposure for the patient and OR staff without the procedures´ outcome and safety being affected.

## Introduction

From the onset of renal colic, urolithiasis patients are at risk of radiation exposure [1]. According to the current European Guidelines, non-contrasted computed tomography (grade of recommendation A, LE 1a) is recommended in patients with acute flank pain suspected of having urolithiasis after initial ultrasound assessment [2]. Therefore, even imaging diagnostics bear a potential radiation exposure for urolithiasis patients. In addition, all interventional treatment options require fluoroscopy and expose patients and OR staff alike to radiation. Considering the current treatment trends favoring ureteroscopic procedures [3], radiation exposure during ureteroscopy is a crucial topic which bears a high potential for optimization [4].

The harming effects of radiation can be subdivided into deterministic and stochastic effects [1, 5]. Notably, cancer-inducing mutations have a stochastic genesis and no cancer-inducing stochastic threshold can be defined. Therefore, the American College of Radiology “supports the ‘as low as reasonably achievable’ (ALARA) concept which urges providers to use the minimum level of radiation needed in imaging exams to achieve the necessary results” [6].

Endourologists can impinge on patients´ radiation exposure over two main determinants: duration of X-ray usage, expressed by the fluoroscopy time (FT), and the applied dose of radiation, expressed by the dose area product (DAP), which is related to the effective dose [1]. There are several approaches reported in the urologic literature to reduce radiation exposure during ureteroscopy which tackle these main determinants. Several studies have shown that increasing surgeon experience leads to reduced FT during ureteroscopy [7, 8]. Moreover, highly standardized ureteroscopy protocols and expert team building have similarly proven to significantly reduce FT [9, 10].

Furthermore, two monocentric studies showed that an increased awareness regarding radiation exposure of the performing endourologist also leads to reduced FT [11, 12]. In a broader sense, this effect could be described as a type of Hawthorne effect, also known as the observer effect [13]. The aim of the present study was to confirm the existing monocentric studies´ results in a multicenter and prospective study design. For this purpose, we recruited six German-speaking tertiary care hospitals and included over 300 patients.

## Materials and methods

### Study design

Overall, 6 study centers (Regensburg/GER, Hamburg/GER, Cologne/GER, Wien/AUT, Münster/GER and Freiburg/GER) participated. Prior to the study´s onset, all surgeons were briefed by the local investigator about the hazards of radiation and on strategies to avoid high doses in endourologic surgery assessed by FT and DAP. This X-ray training contained the strategies of using two monitors with saved pictures, using of tactile feedback, effect of increased surgeon awareness and usage of pulsed fluoroscopy. We included all participating consultants and residents into the X-ray teaching. The X-ray training was standardized using a unified PowerPoint presentation which was held over around 30 min in a course at each study center by the local investigator. Furthermore, a standardized note was placed in every operating theater highlighting the potential study inclusion of RIRS into the present study. This prospective study arm was compared to past consecutive RIRS procedures in each study center, prior to surgeon’s X-ray training. The inclusion criteria were RIRS with laser lithotripsy of kidney stones of any size, informed consent of the patient and prior informed surgeon that fluoroscopy data and operative outcomes will be included into a multicenter study. Results were analyzed using *T* test, chi-squared test, univariate analysis and confirmed in a multivariate regression model.

### Ethical standards

The present study was performed according to the Declarations of Helsinki and was approved by the local ethics committee of each study center (leading committee of the University of Regensburg, Germany, IRB Number 16-101-0064).

### Outcome parameters and patient/procedure characteristics

FT was defined as the primary outcome parameter. To evaluate the procedures outcome and safety, endoscopic stone-free rate (SFR), postureteroscopic lesion scale (PULS), and complications according to modified Clavien–Dindo and SATAVA were used [14–16]. Stone clearance was assessed dichotomously: endoscopic stone free or not stone free. Cases with residual fragments < 1 mm were declared stone free according to the Clinical Research Office of the Endourologic Society (CROES) [17]. Patients’ characteristics comprised age, gender, cumulative stone burden, stone location, and number of stones. Procedure characteristics comprised status of prestenting, status of postoperative stenting, use of ureteral access sheath (UAS), operative time, and surgeon’s experience (< 100/ ≥ 100 procedures).

### Statistical evaluation

Statistical analysis was performed using IBM SPSS Statistics (for Windows version 23.0, IBM Corp, Armonk, NY). In monocentric (study center) analysis, Fisher’s exact test (dichotomous variables) and two-tailed T-Test (metric variables) were performed in a manner of multiple testing. Results of univariate analysis were confirmed in a multivariate regression model. *p *values < 0.05 were considered statistically significant.

## Results

Overall, 303 patients were included in the study and subdivided further into 145 retrospective and 158 prospective patients between 11/2016 and 05/2018. All patients’ characteristics subdivided by anonymized study centers are illustrated in Table 1. Herein, there were no statistically significant differences between the two study arms. We omitted illustrating confidence intervals in *T* tests to simplify the illustration.

**Table 1:** Patients’ characteristics

Characteristics and study center	Retrospective study arm	Prospective study arm	*p *value
Study center 1, number of patients	17	33	N/A
Mean age	54.3	51.9	0.636
Sex (female/male)	6/11	17/16	0.372
Proportion of lower pole stones	35.3%	62.5%	0.082
Mean cumulative stone burden (mm)	11.2	10.2	0.17
BMI (mean)	27.4	29.4	0.233
Number of stones (mean)	1.5	1.7	0.57
Study center 2, number of patients	23	24	N/A
Mean age	49.1	45.2	0.421
Sex (female/male)	8/15	4/20	0.193
Proportion of lower pole stones	56.5%	62.5%	0.770
Mean cumulative stone burden (mm)	10.7	10.2	0.818
BMI (mean)	26.1	28.4	0.146
Number of stones (mean)	1.7	1.6	0.820
Study center 3, number of patients	27	27	N/A
Mean age	44.8	50.9	0.170
Sex (female/male)	15/12	10/17	0.275
Proportion of lower pole stones	70.4%	74.1%	1.0
Mean cumulative stone burden (mm)	10.6	13.7	0.06
BMI (mean)	26.2	28.8	0.114
Number of stones (mean)	2.2	2.9	0.106
Study center 4, number of patients	25	25	N/A
Mean age	56.1	48.9	0.050
Sex (female/male)	9/16	9/16	1.0
Proportion of lower pole stones	48.0%	72.0%	0.148
Mean cumulative stone burden (mm)	10.6	11.5	0.530
BMI (mean)	27.9	28.8	0.577
Number of stones (mean)	1.9	1.8	0.694
Study center 5, number of patients	20	20	N/A
Mean age	44.2	50.5	0.232
Sex (female/male)	6/14	10/10	0.333
Proportion of lower pole stones	65.0%	45.0%	0.341
Mean cumulative stone burden (mm)	10.5	11.8	0.328
BMI (mean)	26.0	27.6	0.281
Number of stones (mean)	N/A	N/A	N/A
Study center 6, number of patients	33	29	N/A
Mean age	52.5	54.8	0.618
Sex (female/male)	15/18	14/15	1.0
Proportion of lower pole stones	81.8%	62.1%	0.096
Mean cumulative stone burden (mm)	12.3	8.6	0.093
BMI (mean)	27.3	24.5	0.11
Number of stones (mean)	1.7	1.4	0.178
Data overall study centers	145	158	N/A
Mean age	50.2	50.6	0.824
Sex (female/male)	59/86	64/94	1.0
Proportion of lower pole stones	62.1%	63.7%	0.812
Mean cumulative stone burden (mm)	11.0	10.9	0.722
BMI (mean)	26.9	27.9	0.125
Number of stones (mean)	1.8	1.9	0.559

Procedures’ characteristics were subdivided accordingly in study centers and are illustrated in Table 2. Herein, statistically significant differences are marked in bold letters. Significant differences were observed in the operative time of study center 1 and 4, as well as in the surgeon’s experience of study center 4. Additionally, study center 6 significantly changed their approach regarding the insertion of a UAS. Notably, all significant changes here could be caused by multiple testing.

**Table 2:** Procedures’ characteristics

Characteristics and study center	Retrospective study arm	Prospective study arm	*p *value
Study center 1, number of patients	17	33	N/A
Prestenting	88.2%	68.8%	0.175
Poststenting	100%	100%	N/A
Ureteral access sheath used	35.3%	51.5%	0.372
Surgeon’s experience ≥ 100	52.9%	57.6%	0.773
Time of surgery (min)	80.6	58.7	**0.003**
Study center 2, number of patients	23	24	N/A
Prestenting	100%	100%	N/A
Poststenting	100%	100%	N/A
Ureteral access sheath used	100%	100%	N/A
Surgeon’s experience ≥ 100	78.3%	70.8%	0.740
Time of surgery (min)	60.2	50.0	0.191
Study center 3, number of patients	27	27	N/A
Prestenting	70.4%	85.2%	0.327
Poststenting	92.6%	92.6%	1.0
Ureteral access sheath used	85.2%	96.3%	0.351
Surgeon’s experience ≥ 100	96.3%	85.2%	0.351
Time of surgery (min)	90.9	88.7	0.821
Study center 4, number of patients	25	25	N/A
Prestenting	100%	96.0%	1.0
Poststenting	100%	100%	N/A
Ureteral access sheath used	36.0%	40.0%	1.0
Surgeon’s experience ≥ 100	84.0%	32.0%	**0.000**
Time of surgery (min)	54.8	78.2	**0.010**
Study center 5, number of patients	20	20	N/A
Prestenting	80.0%	85.0%	1.0
Poststenting	85.0%	90.0%	1.0
Ureteral access sheath used	N/A	N/A	N/A
Surgeon’s experience ≥ 100	65.0%	85.0%	0.273
Time of surgery (min)	90.1	88.9	0.910
Study center 6, number of patients	33	29	N/A
Prestenting	33.3%	44.8%	0.437
Poststenting	81.8%	72.4%	0.544
Ureteral access sheath used	90.9%	65.5%	**0.026**
Surgeon’s experience ≥ 100	78.8%	79.3%	1.0
Time of surgery (min)	68.1	63.3	0.543
Data overall study centers	145	158	N/A
Prestenting	75.2%	78.3%	0.585
Poststenting	92.4%	92.4%	1.0
Ureteral access sheath used	72.8%	69.6%	0.588
Surgeon’s experience ≥ 100	77.9%	67.7%	0.053
Time of surgery (min)	73.5	70.2	0.394

Bold values indicate that statistically significant value of *p* < 0.05

**Fig. 1:**
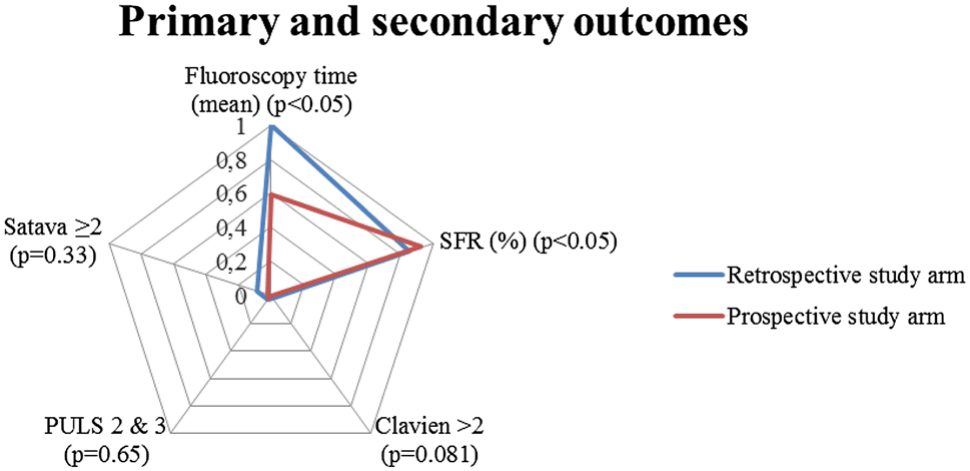
Radar chart, primary and secondary outcomes

The primary and secondary outcomes are illustrated in the radar chart, Fig. 1. Overall, mean FT and DAP (no unit available due to different units of the study centers) were reduced from 130.8 s/560.6 to 77.4 s/357.8 (*p* < 0.05). Endoscopic SFR was improved from 85.5 to 92.4% in the prospective study arm (*p* = 0.04). Complications were not different between the study groups according to the Clavien–Dindo (*p* = 0.081), PULS (*p* = 0.651) and SATAVA (*p* = 0.334) classifications.

In a linear regression model, BMI had a significant impact on DAP (HR 27.4; *p* = 0.003), while BMI had no significant impact on FT (HR 0.01; *p* = 0.99). The number of stones, cumulative stone burden, status of PULS, and stone location had no significant impact on FT. Operative time significantly prolonged FT (HR 1.83; *p* > 0.001). In the retrospective study group, FT was significantly prolonged (HR 53.45; *p* > 0.001). Poststenting significantly prolonged FT (HR 70.23; *p* = 0.009). Interestingly, surgeon experience (> 100 procedures) did not significantly reduce FT.

Significant univariate parameters were confirmed in a multivariate model, revealing X-ray training (synonymously “study group”) to be radiation protective (OR − 44.4, *p* = 0.001); while, an increased FT was confirmed for postprocedural ureteral stent placement (OR 68.6, *p* = 0.028), dusting instead of fragmenting (OR 92.9, *p* < 0.001), UAS usage (OR 59.5, *p* < 0.001) and intraoperative complications according to SATAVA classification (OR 26.6, *p* = 0.022), (please see Table 3).

**Table 3:** Logistic regression model

Analyzed parameter	Odds ratio	CI 95%	*p *value
Study group	− 44.4	[− 70.2; − 18.6]	0.001
Poststenting	68.6	[7.5; 129.6]	0.028
Dusting vs. fragmenting	92.9	[65.1; 120.7]	0.00
Ureteral access sheath used	59.5	[29.1; 89.9]	0.00
SATAVA > 0	26.6	[3.8; 49.5]	0.022

## Discussion

There is increasing concern about the radiation exposure of urolithiasis patients during diagnosis and treatment [1]. Therefore, urologists should aim to treat lithiasis patients according to the ALARA-based principles [6]. During the last decade, (flexible) ureteroscopy has been gaining importance as the most favored treatment modality for renal and ureteral stones, continuously pushing shock wave lithotripsy into the background [3, 18]. However, ureteroscopy and especially flexible ureteroscopy may be linked to significant radiation exposure for the patient and OR staff [4]. Therefore, radiation exposure during flexible ureteroscopy should be kept as low as reasonably achievable.

In the present work, we evaluated the impact of preoperative surgeon briefing on FT, DAP and surgical outcomes in a multicenter study of applied X-ray exposure. Prospective results were compared to retrospective baseline data of each study center. Overall, mean FT and DAP were reduced from 130.8 s/560.6 to 77.4 s/357.8 (*p* < 0.05), respectively. This translates into a FT reduction of 40.8%. In the prospective study arm, we demonstrated that surgical outcomes, such as stone clearance and complications according to SATAVA and Clavien–Dindo classifications, showed no significant variation to the baseline data or were even improved (stone clearance from 85.5 to 92.4% in the prospective study arm, *p* = 0.04). Therefore, reduction of radiation exposure is safe and does not decrease quality of treatment. In a multivariate analysis, we showed the prospective study group (synonymously increased surgeon awareness) to be highly radiation protective (OR − 44, *p* = 0.001, see Table 3).

Ngo et al. [11] first described a significant FT reduction effect by providing surgeons with feedback on his or her applied FT in ureteroscopy, similar to the present study’s results. In this monocentric study, endourologists obtained feedback about their own applied FT during ureteroscopy as well as the applied FT of their colleagues. The FT baseline data were based on an initial 9-month study period in which FT was recorded. Overall, 311 ureteroscopic procedures were evaluated. Stone location differed in one-quarter of kidney stones and three-quarters of ureteral stones. In multivariate regression analysis, female gender, distal ureteral stones and surgeon feedback were identified as independent predictors for reduced FT. Overall, Ngo and colleagues showed a 24% FT reduction from 2.74 to 2.08 min (*p* = 0.002) by providing surgeon’s feedback. Taking this into account, we surpassed these results in our study by a 40.8% FT reduction in a multicenter study design.

In 2014, Weld et al. [12] published a comparable work. In their monocentric study, investigators evaluated the impact of “Safety, Minimization and Awareness Radiation Training (SMART)” on the FT of 4 urologic residents during 202 ureteroscopic procedures [12]. Training details were not specified; however, SMART reduced FT by 56% when comparing baseline FT data (FT of 102 s) to post-SMART FT data (FT of 45 s; *p* < 0.001). Herein, the proportion of renal stones was 45% (92 procedures). Weld et al. showed a greater reduction in FT compared to our study and more favorable results when comparing the absolute values of FT. However, Weld et al., evaluated FT in ureteroscopic procedures for ureteral (55%) and renal (45%) stones. In our work, solely RIRS procedures including laser lithotripsy for renal stones were included—and RIRS has been associated with increased radiation exposure compared to ureteroscopy for ureteral stones, as in the study by Weld et al. [12].

In multivariate analysis, we showed postprocedural JJ placement, insertion of a UAS, and complications according to SATAVA > 0 to be significantly associated with an increased FT. All of these factors seem to increase radiation exposure parameters and similar results have been presented in the literature [11, 12]. Nevertheless, we should critically scrutinize these radiation increasing factors. The current EAU guidelines on interventional treatment for urolithiasis link UAS placement to decreased intrarenal pressures, improved access to the upper urinary tract, enhanced vision and reduced operative time [19]. Potential ureteral damage is mentioned as the sole disadvantage, while increased radiation exposure is not. However, urologists should be aware of increased radiation exposure during UAS placement. On the other hand, the guideline refers to several RCTs which found that routine postprocedural JJ placement after uncomplicated ureteroscopy is not necessary [19]. The 92.4% rate of poststented patients in all participating centers of our study represents a divergent clinical practice, which might be caused by requirements of the German and Austrian health care systems.

Apart from the evaluated “awareness effect”, there are other simple approaches to reduce radiation exposure during (flexible) ureteroscopy. The standardization of ureteroscopic procedures appears to be an important approach. In a previously published study, part of our group presented the impact of an ultralow fluoroscopy protocol on applied X-ray usage [10]. In flexible ureteroscopy, FT was reduced from 167.7 s in 2009 to 7.4 s in 2015 (*p* < 0.001); while, surgical outcomes and complications showed no statistically significant differences. Greene et al. [9] showed a similar reduction in FT from 86 to 16 s using a standardized protocol. Hsi et al. [20] evinced that even fluoroless (flexible) ureteroscopy is feasible using a highly standardized protocol in the majority of 162 consecutive ureteroscopic procedures.

Another important potentially radiation reducing factor is the surgeon’s experience. Several studies have shown the radiation-protective effect of increased surgeon experience on FT [7, 11]. However, it is not possible to differentiate the impact of standardized protocols and of an increased surgeon’s experience reliably [10]. Interestingly, we could not show a radiation-protective effect of increased surgeon experience in our present work. The reason may be that in our study, surgeons of the prospective study arm were “biased” regarding the FT independently of their own experience. Consequently, an increased awareness seems to be a more important factor on FT than surgeon experience is.

Our results identified obesity as a parameter significantly increasing the applied radiation dose, reflected by a significantly increased DAP (HR = 27.37, *p* = 0.003). On the other hand, obesity did not raise FT significantly (HR = 0.01, *p* = 0.99). The raised radiation dose effect in obese patients is in accordance with existing evidence of phantom and clinical studies [21, 22].

In the future, robot-assisted flexible ureteroscopy might reduce the radiation exposure to the OR staff, while the patient himself will have no benefit from this effect [23, 24].

Each study has its own limitations. In the current study design, we did not evaluate the X-ray settings of each study center. On the other hand, we recommended the application of pulsed fluoroscopy in our standardized X-ray training. The effect of the probably raised application of pulsed fluoroscopy in the prospective study arm on the DAP could not be differed in the present study design. However, the effect on our main outcome parameter FT should not be influenced by this.

In the present study, we demonstrated increased surgeon awareness to be highly radiation protective in RIRS treatment. RIRS performing endourologists should be routinely briefed about their own radiation reducing impact. Moreover, highly standardized protocols, increased surgeon’s experience and expert team building are other effective strategies to help reduce radiation exposure in patients and OR staff during RIRS.

## Conclusions

The stochastic effects of radiation exposure sway physicians to reduce applied radiation in diagnostics and treatment whenever possible. RIRS treatment may require significant radiation exposure in lithiasis patients. In the present multicenter study, we demonstrated increased surgeon awareness regarding X-ray exposure to be highly radiation protective. Therefore, endourologists performing RIRS should be sensitized about their important impact on applied radiation whenever performing RIRS. Such wide-ranging effects might be even achieved by a simple mean, as presented in our study.

## Abbreviations:

FT: Fluoroscopy time
DAP: Dose area product
RIRS: Retrograde intrarenal surgery
SFR: Stone-free rate

## References

[1] Chen TT, Wang C, Ferrandino MN, Scales CD, Yoshizumi TT, Preminger GM, Lipkin ME (2015). Radiation exposure during the evaluation and management of nephrolithiasis. J Urol.

[2] Turk C, Knoll T, Petrik A, Sarica K, Skolarikos A, Straub M, Seitz C (2015) EAU guidelines on urolithiasis 2015. https://uroweb.org/guideline/urolithiasis/

[3] Oberlin DT, Flum AS, Bachrach L, Matulewicz RS, Flury SC (2015). Contemporary surgical trends in the management of upper tract calculi. J Urol.

[4] Lipkin ME, Wang AJ, Toncheva G, Ferrandino MN, Yoshizumi TT, Preminger GM (2012). Determination of patient radiation dose during ureteroscopic treatment of urolithiasis using a validated model. J Urol.

[5] Scott BR (2006). Stochastic thresholds: a novel explanation of nonlinear dose-response relationships for stochastic radiobiological effects. Dose-response Publ Int Hormesis Soc.

[6] American College of Radiology (ACR) (2011) ACR Statement on FDA radiation reduction program. https://www.acr.org/Advocacy-and-Economics/ACR-Position-Statements/FDA-Radiation-Reduction-Program

[7] Ritter M, Siegel F, Krombach P, Martinschek A, Weiss C, Hacker A, Pelzer AE (2013). Influence of surgeon's experience on fluoroscopy time during endourological interventions. World J Urol.

[8] Weld LR, Nwoye UO, Knight RB, Baumgartner TS, Ebertowski JS, Stringer MT, Kasprenski MC, Weld KJ (2015). Fluoroscopy time during uncomplicated unilateral ureteroscopy for urolithiasis decreases with urology resident experience. World J Urol.

[9] Greene DJ, Tenggadjaja CF, Bowman RJ, Agarwal G, Ebrahimi KY, Baldwin DD (2011). Comparison of a reduced radiation fluoroscopy protocol to conventional fluoroscopy during uncomplicated ureteroscopy. Urology.

[10] Hein S, Schoenthaler M, Wilhelm K, Schlager D, Vach W, Wetterauer U, Miernik A (2017). Ultralow radiation exposure during flexible ureteroscopy in patients with nephrolithiasis-how far can we go?. Urology.

[11] Ngo TC, Macleod LC, Rosenstein DI, Reese JH, Shinghal R (2011). Tracking intraoperative fluoroscopy utilization reduces radiation exposure during ureteroscopy. J Endourol Endourol Soc.

[12] Weld LR, Nwoye UO, Knight RB, Baumgartner TS, Ebertowski JS, Stringer MT, Kasprenski MC, Weld KJ (2014). Safety, minimization, and awareness radiation training reduces fluoroscopy time during unilateral ureteroscopy. Urology.

[13] McCarney R, Warner J, Iliffe S, van Haselen R, Griffin M, Fisher P (2007). The Hawthorne effect: a randomised, controlled trial. BMC Med Res Methodol.

[14] de la Rosette JJ, Opondo D, Daels FP, Giusti G, Serrano A, Kandasami SV, Wolf JS, Grabe M, Gravas S (2012). Categorisation of complications and validation of the Clavien score for percutaneous nephrolithotomy. Eur Urol.

[15] Schoenthaler M, Wilhelm K, Kuehhas FE, Farin E, Bach C, Buchholz N, Miernik A (2012). Postureteroscopic lesion scale: a new management modified organ injury scale–evaluation in 435 ureteroscopic patients. J Endourol Endourol Soc.

[16] Tepeler A, Resorlu B, Sahin T, Sarikaya S, Bayindir M, Oguz U, Armagan A, Unsal A (2014). Categorization of intraoperative ureteroscopy complications using modified Satava classification system. World J Urol.

[17] de la Rosette J, Denstedt J, Geavlete P, Keeley F, Matsuda T, Pearle M, Preminger G, Traxer O (2014). The clinical research office of the endourological society ureteroscopy global study: indications, complications, and outcomes in 11,885 patients. J Endourol Endourol Soc.

[18] Miernik A, Wilhelm K, Ardelt P, Bulla S, Schoenthaler M (2012). Modern urinary stone therapy: is the era of extracorporeal shock wave lithotripsy at an end?. Der Urologe Ausg A.

[19] Turk C, Petrik A, Sarica K, Seitz C, Skolarikos A, Straub M, Knoll T (2016). EAU Guidelines on interventional treatment for urolithiasis. Eur Urol.

[20] Hsi RS, Harper JD (2013). Fluoroless ureteroscopy: zero-dose fluoroscopy during ureteroscopic treatment of urinary-tract calculi. J Endourol Endourol Soc.

[21] Shin RH, Cabrera FJ, Nguyen G, Wang C, Youssef RF, Scales CD, Ferrandino MN, Preminger GM, Yoshizumi TT, Lipkin ME (2016). Radiation dosimetry for ureteroscopy patients: a phantom study comparing the standard and obese patient models. J Endourol Endourol Soc.

[22] Hsi RS, Zamora DA, Kanal KM, Harper JD (2013). Severe obesity is associated with 3-fold higher radiation dose rate during ureteroscopy. Urology.

[23] Muller PF, Schlager D, Hein S, Bach C, Miernik A, Schoeb DS (2018). Robotic stone surgery—current state and future prospects: a systematic review. Arab J Urol.

[24] Saglam R, Muslumanoglu AY, Tokatli Z, Caskurlu T, Sarica K, Tasci AI, Erkurt B, Suer E, Kabakci AS, Preminger G, Traxer O, Rassweiler JJ (2014). A new robot for flexible ureteroscopy: development and early clinical results (IDEAL stage 1–2b). Eur Urol.

